# Dietary Supplementation with Hesperidin and Rosmarinic Acid Improves Meat Quality, Modulates Gut Microbiota, and Enhances Antioxidant Capacity in Finishing Pigs

**DOI:** 10.3390/microorganisms14071518

**Published:** 2026-07-12

**Authors:** Jianmin Wu, Wenxuan Zhao, Xuedong Ding, Shiyi Tian, Jing Wang

**Affiliations:** 1 National Center for International Research on Animal Gut Nutrition, Jiangsu Key Laboratory of Gastrointestinal Nutrition and Animal Health, Laboratory of Gastrointestinal Microbiology, National Experimental Teaching Demonstration Center of Animal Science, College of Animal Science and Technology, Nanjing Agricultural University, Nanjing 210095, China; 2020205028@stu.njau.edu.cn (J.W.); 2020105062@stu.njau.edu.cn (W.Z.); 2021205031@stu.njau.edu.cn (X.D.); 2 College of Animal Science and Technology, Jiangxi Agricultural University, Nanchang 330045, China

**Keywords:** meat quality, hesperidin, rosmarinic acid, gut microbiota, amino acids, finishing pigs

## Abstract

Enhancing meat quality and optimizing pork-free amino acid profiles can boost the industry competitiveness of pork. Plant-extracted phenolic substances have beneficial effects on meat quality. This study evaluated the effects of dietary supplementation with hesperidin, rosmarinic acid, and their combination on meat quality in finishing pigs. Twenty-four pigs were divided into four dietary treatment groups: CON (basal diet), HES (basal diet supplemented with 600 mg/kg hesperidin), RA (basal diet supplemented with 40 mg/kg rosmarinic acid), and HES-RA (basal diet supplemented with 300 mg/kg hesperidin and 20 mg/kg rosmarinic acid). After 90 days, pigs were slaughtered and sampled following a 12 h fast. Compared with the CON group, the HES-RA group pigs exhibited a significant increase in pH45 min and meat color a*, and had a decrease in meat color L* and drip loss. The combined HES-RA supplementation significantly increased the activities of glutathione peroxidase, total antioxidant capacity, and total superoxide dismutase, while reducing malondialdehyde. Furthermore, the contents of arginine and leucine were elevated in the muscle and serum. This combination treatment also modulated the ileal microbiota composition, notably enriching short-chain fatty acid-producing genera such as *Veillonella* and *Mitsuokella*, and increased the concentrations of acetate, propionate, and butyrate in the ileum. Furthermore, the combined supplementation significantly elevated the muscle mRNA expression of *GLUT4*, *mTOR*, *S6K1*, and *4E-BP1*. Collectively, the hesperidin and rosmarinic acid supplementation was associated with enhanced meat quality, along with improved antioxidant capacity, modulation of gut microbiota and SCFA production, and upregulation of mTOR-associated gene expression.

## 1. Introduction

The global demand for high-quality pork continues to rise, with consumers increasingly prioritizing sensory characteristics, nutritional value, and food safety. Multiple variables contribute to meat quality as a multifaceted phenotype, notably the level of intramuscular fat, the balance of fatty acids, the capacity of the antioxidant defense system, and the profile of amino acids [1]. In modern swine production, genetic selection has primarily focused on improving growth rate and lean meat percentage, which has often coincided with a decline in pork quality attributes such as flavor, juiciness, and oxidative stability [1]. Consequently, nutritional strategies have emerged as effective and feasible means to enhance meat quality in finishing pigs.

The prohibition of antibiotic growth promoters in the feed industry has accelerated the search for natural and safe alternatives. Plant-derived phenolic compounds have attracted considerable research interest due to their remarkable biological activities, including antioxidant, anti-inflammatory, and antibacterial properties [2,3,4]. Hesperidin and rosmarinic acid are polyphenolic compounds commonly found in plant extracts. Hesperidin, a citrus flavonoid, improves meat quality in broilers by enhancing oxidative stability and water-holding capacity [5,6], but its poor bioavailability necessitates microbial conversion in the gut to release active forms [7]. Rosmarinic acid, a phenolic compound from rosemary, is known for its strong antioxidant capacity and has been reported to regulate lipid metabolism and shape the gut microbiota in pigs [1,8,9]. Our previous studies demonstrated that combined supplementation of these two compounds improved meat quality in broilers and intestinal health in finishing pigs [10,11,12]. Thus, we hypothesized that dietary supplementation with a combination of hesperidin and rosmarinic acid could improve meat quality, and optimize the amino acid profile of pork in finishing pigs. To test this hypothesis, we evaluated carcass characteristics, meat quality parameters, free amino acid profiles in serum and muscle, antioxidant capacity in muscle tissue, gut microbiota composition, SCFA concentration and mRNA expression levels in finishing pigs fed a diet supplemented with this combination.

## 2. Materials and Methods

### 2.1. Chemicals

Hesperidin (purity ≥ 95%, batch no. HES20230512, crystalline powder) and rosmarinic acid (purity ≥ 98%, batch no. RA20230428, crystalline powder) were purchased from Yuning Biotechnology (Taiyuan, China). The BCA protein assay kit (catalog no. BL521A) was obtained from Biosharp (Hefei, China). Commercial kits were procured from the Nanjing Jiancheng Bioengineering Institute (Nanjing, China) for catalase (CAT, A007-1), glutathione peroxidase (GSH-Px, A005-1), total antioxidant capacity (T-AOC, A015-2), malondialdehyde (MDA, A003-1), and total superoxide dismutase (T-SOD, A001-3). The additional chemicals employed were of analytical quality, and they were sourced from Yuanye Biotechnology (Shanghai, China).

### 2.2. Animal Experimental Design

A total of 24 healthy barrows (Duroc × Landrace × Yorkshire) with an average initial body weight of 40.00 ± 2.80 kg were assigned to four dietary treatments using a randomized complete block design. The pigs were first grouped into blocks based on similar body weight to minimize initial weight variation. Within each block, they were then randomly allocated to the four treatment groups, resulting in six replicates (pigs) per group. The experimental diets consisted of a basal diet (CON); the basal diet supplemented with 600 mg/kg hesperidin (HES); the basal diet with 40 mg/kg rosmarinic acid (RA); and the basal diet containing 300 mg/kg hesperidin and 20 mg/kg rosmarinic acid (HES-RA). The dosage of hesperidin (600 mg/kg) and rosmarinic acid (40 mg/kg) for the individual treatment groups was selected based on previous studies in swine and our own preliminary trials, which indicated that these levels were effective and safe [11,12,13,14]. The combination (HES-RA) dose was set at half of each individual dose to evaluate whether lower amounts of each compound, when used in combination, could still produce comparable or enhanced responses, potentially offering a more cost-effective strategy. The basal diet was prepared following the NRC (2012) [15] nutrient recommendations, with the details listed in Table 1. The supplemented diets were prepared fresh in weekly batches, stored in airtight containers protected from light at room temperature, and used within 7 days to minimize potential degradation of the phenolic compounds. Hesperidin and rosmarinic acid were incorporated into the basal diet through stepwise mixing to ensure uniform distribution: each compound was first premixed with a small amount of basal diet (1 kg), then progressively diluted with larger volumes until the full batch was homogenized. The target inclusion levels were verified gravimetrically during preparation. While the purity of the test compounds was certified by the supplier (≥95% for hesperidin, ≥98% for rosmarinic acid), direct analytical quantification of residual compound levels in the finished feed was not performed and should be considered in future studies. The nutrient composition of the experimental diet was analyzed according to the methods established by the Association of Official Analytical Chemists (AOAC, 2005) [16]. Calculations were based on the Feed Database in China (https://www.chinafeeddata.org.cn) [17]. According to the procedures outlined by AOAC (2005), the quantification of crude protein was performed using Kjeldahl’s method (AOAC 954.01), and the ether extract was assessed via Soxhlet extraction (AOAC 920.39). Digestible energy and additional nutrients were computed based on the Feed Database in China (2020) guidelines.

The 90-day feeding trial was conducted under temperature-controlled conditions (26 ± 2 °C), with all pigs housed individually in pens providing ample space (1.0 m × 2.1 m) and free access to food and water. Specifically, the diet was adjusted at each of three body weight phases: from initial weight to 50 kg, from 50 kg to 80 kg, and from 80 kg to slaughter weight, to ensure nutritional requirements were precisely met throughout the entire 90-day period. On the morning of day 90, following a 12 h fast, the body weight of each pig was recorded. After the trial, all pigs were transported 48 km to a commercial abattoir and underwent an overnight fast prior to euthanasia. The euthanasia protocol involved immobilizing the pigs through an electrical stunning (250 V, 0.5 A, for 5–6 s), followed by exsanguination, according to ethical guidelines to ensure humane treatment. Growth performance parameters, including average daily gain (ADG), average daily feed intake (ADFI), and feed-to-gain ratio (F/G), were recorded throughout the trial. Detailed values are provided in Supplementary Material Table S1 (reproduced from our previous study [11]).

### 2.3. Blood Samples

Venous blood samples were collected from all animals after a 12 h overnight fast to standardize postprandial conditions and minimize dietary influences on metabolic parameters. To control for potential diurnal variation, all samples were obtained within a consistent morning time window (08:00–10:00) on the sampling day. To prevent hemolysis, blood was collected using vacuum tubes and handled gently throughout the process; forceful transfer or agitation of the tubes was strictly avoided. At room temperature, whole blood was centrifuged at 1800× *g* for 10 min; the resulting serum was aliquoted into cryovials and then stored at −80 °C for subsequent testing.

Following slaughter and evisceration at a commercial abattoir, the carcasses were placed in commercial refrigerators and maintained at a constant 4 °C throughout the post-mortem inspection process.

### 2.4. Carcass Characteristics Measurements

After slaughtering, slaughter performance was determined based on the relevant requirements in the Chinese Agricultural Standard (NY/T 825-2004) [18]. Briefly, hot carcass weights were measured to calculate carcass yield. Carcass straight length and carcass slant length were measured with a soft ruler according to the previous method (Deep Learning-Based Automated Approach for Determination of Pig Carcass Traits). The thickness of the subcutaneous fat layer was measured at three anatomical sites—the thickest point of the shoulder along the dorsal midline, the last rib, and the lumbar–sacral junction—using vernier calipers, and the mean backfat thickness was subsequently calculated. The Longissimus lumborum muscle was sampled at the level of the 12th rib to standardize the measurement of eye muscle area (also known as rib-eye area) and evaluate meat quality parameters. The remaining muscle samples were stored at −80 °C for amino acid profile and gene expression analysis. The right side of the carcass was dissected to separate lean meat, fat, bone, and skin, and their masses were then measured. The following formulas were used for calculations:

Leanness (%) = (Lean mass/carcass mass) × 100%
Fatness (%) = (Fat mass/carcass mass) × 100%
Bone rate (%) = (Bone mass/carcass mass) × 100%
Skin rate (%) = (Skin mass/carcass mass) × 100%

### 2.5. Meat Quality Measurement

The meat quality analysis was conducted on Longissimus lumborum muscle samples stored at 4 °C, following a sequential workflow that progressed from analysis of fresh samples to cooked sample evaluation. The procedures for assessing pH, water-holding capacity (WHC), meat color, cooking loss, and shear force are described below, with reference to established methodologies [19].

The pH of the fresh muscle was measured at 45 min and 24 h post-slaughter using a portable pH meter (F3-standard, Mettler Toledo, Columbus, OH, USA). Prior to each measurement session, the pH meter was calibrated with standard buffers (pH 4.01 and 7.00) using a two-point calibration method. An automatic temperature compensation (ATC) probe was employed during both calibration and measurement to ensure accuracy across varying sample temperatures. WHC was assessed by measuring drip loss. Approximately 10 g of muscle was suspended in a sealed plastic bag at 4 °C for 48 h. Drip loss (%) was determined based on the weight reduction in the samples following suspension, with initial and final weights measured. A tristimulus colorimeter (CR-410, Konica Minolta Sensing, Osaka, Japan) was used to measure color parameters on the surface of fresh muscle samples. Measurements were performed under standardized conditions: Illuminant D65, a 2° Standard Observer, and an 8 mm aperture size. The color values recorded were lightness (L), redness (a), and yellowness (b*).

After the fresh sample analyses, the remaining muscle specimens were vacuum-packed and subjected to a 70 °C water bath until their internal geometric temperature reached 70 °C. The vacuum sealing prevented water ingress and minimized evaporative loss during heating. All samples were processed across seven independent, fully randomized cooking batches to mitigate potential batch effects. Immediately after cooking, the internal temperature of each sample was verified using a thermocouple thermometer. Samples failing to achieve the target endpoint temperature of 70 ± 0.5 °C were discarded. Across all cooking batches, a total of three samples (with no more than one from any single treatment group) were discarded for failing to meet the target endpoint temperature and were excluded from the cooking loss and shear force analysis. Cooking loss was determined by measuring the weight change before and after cooking and expressing the weight loss as a percentage of the initial sample weight.

Shear force, an objective measure of tenderness, was evaluated on the cooked samples. Cylindrical cores (n = 4 per sample), with a mean weight of 35.2 ± 2.5 g, were taken parallel to the muscle fiber orientation from each cooked sample. Each core was sheared once using a Warner–Bratzler blade attached to a TA.XT Plus Texture Analyzer (fitted with a 25 kg load cell) or a digital shear meter (HP609, Jinan Hengpin Electromechanical, Jinan, Shandong, China). The mean of the four measurements was recorded as the shear force value for the sample.

### 2.6. Determination of Amino Acid Profiles

Sample preparation and acid hydrolysis

Excess peripheral muscle membrane was removed from the Longissimus lumborum muscle samples, which were then diced and homogenized to a fine consistency by passing through a meat grinder. Muscle samples (1 ± 0.0001 g) were accurately weighed using an analytical balance, transferred to hydrolysis tubes, and treated with 14 mL of 6 mol/L hydrochloric acid (HCl). The air in the tubes was displaced using a nitrogen blowing instrument, and the tubes were then vacuum-sealed. Hydrolysis was performed in a constant-temperature forced-air drying oven (DHG-9245A, Yiheng, Shanghai, China) at 103 °C for 24 h. The cooled hydrolysate was transferred completely into a 50 mL volumetric flask, after which ultrapure water was added to reach the calibration mark.

Deproteinization and filtration

For serum samples, deproteinization was achieved by adding 120 mg/mL of salicylic acid to the serum matrix. The mixture was mixed using a vortex mixer for 1 min, then left to stand on ice for 20 min. Subsequently, the pH of the reaction system was adjusted to 7.0–7.5 using 2 mol/L lithium hydroxide (LiOH) to stabilize amino acids. At 4 °C, the solution was centrifuged at 12,000× *g* for 20 min using an Eppendorf 5425 centrifuge (Hamburg, Germany), and the resulting supernatant was collected for subsequent analysis.

Amino acid analysis

For both muscle and serum samples, 1 mL of the processed solution was aspirated, subjected to reduced-pressure drying (Rotary Evaporator R-300, BUCHI, Flawil, Switzerland), and reconstituted in 1 mL of 0.1 mol/L sodium citrate buffer (pH 2.2). The solution was passed through a nylon membrane with a pore size of 0.22 μm (Millipore, Burlington, MA, USA) to remove any particulate impurities that might be present. Amino acid composition was determined using a Model LA8080 automatic amino acid analyzer (Hitachi, Tokyo, Japan) equipped with a lithium-based ion-exchange column (4.6 × 60 mm, 3 μm).

### 2.7. Determination of Antioxidant Properties

Samples (0.1 g each) of the Longissimus lumborum muscle were weighed and placed into centrifuge tubes containing 900 μL of PBS. After homogenization for 1 min and centrifugation (4 °C, 10,000× *g*, 15 min), the supernatants were harvested for total protein quantification with the BCA protein assay kit (Biosharp, Hefei, Anhui, China), as well as for measurement of CAT, GSH-Px, T-AOC, T-SOD, and MDA levels using their corresponding commercial assay kits.

### 2.8. Microbiological Analysis of the Ileum Digesta

An ileal digesta sample (0.3 g) was accurately weighed for the extraction of total DNA from the microbiota. Total DNA was isolated using the Power Soil DNA Isolation Kit (MoBio Laboratories, Carlsbad, CA, USA) according to the manufacturer’s instructions. The concentration and quality of the extracted DNA were determined using a NanoDrop 2000 spectrophotometer (Thermo Fisher Scientific, Waltham, MA, USA). The V3-V4 region of the bacterial 16S rRNA gene was amplified using primers 338F (5′-ACTCCTACGGGAGGCAGCAG-3′) and 806R (5′-GGACTACHVGGGTWTCTAAT-3′). Following PCR with 27 cycles (95 °C for 30 s, 55 °C for 30 s, and 72 °C for 45 s) after initial denaturation at 95 °C for 3 min and before a final 10 min extension at 72 °C, the products were purified by 2% agarose gel electrophoresis, recovered using the AxyPrep Kit (Axygen, Union City, CA, USA), and quantified on a QuantiFluor-ST (Promega, Madison, WI, USA). Equalized samples were then sequenced on the Illumina MiSeq platform (San Diego, CA, USA). Raw reads were deposited in GeneBank (NCBI; PRJNA1063182).

After assembly of overlapping paired-end reads, low-quality sequences were removed using the following criteria: average quality score < 20, length < 200 bp, or the presence of ambiguous bases and primer mismatches. Chimeras were detected and filtered with UCHIME. Clustering of operational taxonomic units (OTUs) was performed using Usearch (v7.0.1090) at a 97% sequence similarity threshold. Taxonomic annotation was performed using the RDP classifier (v2.2) against the Silva 16S rRNA database (Release 132), with a confidence threshold set at 70%. To correct for differences in sequencing depth among samples, α-diversity indices (including the Shannon, Simpson, ACE, and Chao indices) were estimated using the Mothur software (v1.30.2) based on the rarefied OTU table. Community compositional differences were visualized by principal coordinate analysis (PCoA) on Bray–Curtis distances and tested for significance with ANOSIM (Qiime v1.9.1).

### 2.9. Determination of SCFAs

Ileal digesta (0.2 g) was weighed out into a centrifuge tube, and 0.8 mL of double-distilled water was added. After homogenization by vortexing, the suspension was centrifuged (12,000× *g*, 10 min, 4 °C). The resulting supernatant was transferred to a fresh tube and mixed with 0.12 mL of 25% (*w*/*v*) metaphosphoric acid containing crotonic acid (1.25 mg/mL) as the internal standard. Following overnight precipitation of proteins at −20 °C, the sample was centrifuged again under the same conditions. Finally, the supernatant was passed through a 0.22 μm membrane filter into an autosampler vial.

SCFA analysis was performed on a Shimadzu GC-14B gas chromatograph (Shimadzu, Kyoto, Japan) fitted with an FID and a polar DB-FFAP capillary column (30 m × 0.25 mm × 0.25 μm; Agilent, Santa Clara, CA, USA). Nitrogen served as the carrier gas at a constant flow of 1.0 mL/min. The injector and detector were maintained at 250 °C and 280 °C, respectively. The oven temperature program was 60 °C for 1 min, ramp to 180 °C at 10 °C/min (hold 5 min), then ramp to 230 °C at 20 °C/min (hold 2 min). One microliter of sample was introduced in split mode (split ratio 10:1). Individual SCFAs (acetate, propionate, butyrate, isobutyrate, valerate, isovalerate) were quantified by external standard calibration and corrected using crotonic acid as the internal standard.

### 2.10. Quantitative Real-Time PCR

Total RNA was extracted from Longissimus lumborum muscle tissue using Trizol reagent (Waltham, MA, USA). The concentration and purity of the extracted RNA were assessed using a NanoDrop 1000 spectrophotometer (Thermo Fisher Scientific, Waltham, MA, USA). High-quality RNA was reverse transcribed to cDNA using a reverse transcription kit (Agibio Bioengineering Co., Ltd., Changsha, Hunan, China). Quantitative PCR (qPCR) was performed using a fluorescent quantitative PCR instrument. The reaction conditions were based on previously published protocols [20]. The housekeeping gene GAPDH served as the internal control. Relative gene expression was calculated by the 2^−ΔΔCt^ method [21]. Primer sequences are provided in Supplementary Material Table S2. The assay followed the methodology described by a previous study [22].

### 2.11. Statistical Analysis

Statistical analysis was performed using SPSS software (version 22.0, IBM, Armonk, NY, USA). A general linear mixed model was employed to analyze carcass trait-related indicators, as carcass weight varied significantly across individuals and was included as a covariate to control for its confounding effect. Treatment group (CON, HES, RA, HES-RA) was included as a fixed effect. Non-significant terms were removed stepwise from the model using approximate F-ratio tests (*p* < 0.05). From the final model, predicted means and standard errors were obtained, and pairwise comparisons among groups were performed with the least significant difference (LSD) test (α = 0.05). The effects of treatment on the remaining variables were assessed using one-way analysis of variance (ANOVA), and pairwise comparisons between groups were performed with Tukey’s post hoc test. Results were presented as mean ± standard error of the mean (SEM), and *p* < 0.05 was considered statistically significant.

## 3. Results

### 3.1. Carcass Traits

Table 2 presents the carcass traits of finishing pigs under different dietary treatments. Relative to the CON group, the HES-RA group showed higher carcass weight, carcass oblique length, and lean meat percentage (*p* < 0.05), along with lower backfat depth and fat rate (*p* < 0.05). The HES and RA groups also displayed greater lean meat rate and reduced backfat depth compared with the CON group (*p* < 0.05).

### 3.2. Meat Quality

The dietary treatments affected several meat quality traits in finishing pigs, as summarized in Table 3. Pigs in the HES-RA group demonstrated significantly higher pH_45min_ and meat color a* values, along with significantly lower L* values and reduced drip loss compared to the CON group (*p* < 0.05). In contrast, neither the HES nor the RA group showed significant differences in meat quality traits and cooking loss relative to the CON group (*p* > 0.05).

### 3.3. Antioxidant Properties

**Figure 1:**
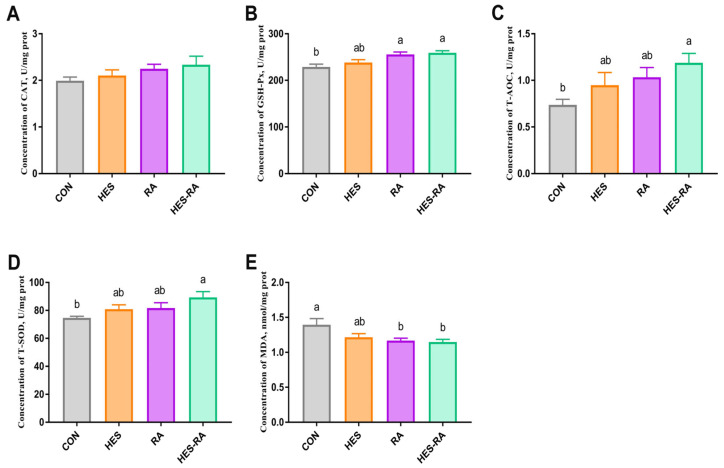
Antioxidant properties of the Longissimus lumborum muscle. (**A**) CAT level. (**B**) GSH-Px level. (**C**) T-AOC level. (**D**) T-SOD level. (**E**) MDA level. Data are presented as the mean ± standard error of the mean (SEM) *(n* = 6). A significant difference (*p* < 0.05) among different groups is indicated by different lowercase letters. CON = control; HES = hesperidin; RA = rosmarinic acid; HES-RA = hesperidin + rosmarinic acid.

As illustrated in Figure 1, the antioxidant capacity of the Longissimus lumborum muscle was significantly influenced by dietary treatments. Relative to the CON group, significantly higher activities of GSH-Px, T-AOC, and T-SOD, and a marked decrease in MDA concentration, were observed in the HES-RA group (*p* < 0.05). Elevated GSH-Px activity and lower MDA levels were likewise detected in the RA group compared with the CON group (*p* < 0.05). Antioxidant parameters were not significantly different between the HES and CON groups (*p* > 0.05).

### 3.4. The Profiles of Free Amino Acids

The free amino acid profile of the Longissimus lumborum muscle is shown in Table 4. Compared with the CON group, the levels of lysine, methionine, isoleucine, leucine, phenylalanine, threonine, alanine, aspartic acid, glutamic acid, arginine, glycine, serine, tyrosine, flavor amino acids, total essential amino acids, total non-essential amino acids, and total amino acids were significantly increased (*p* < 0.05) in the HES-RA group. Additionally, the contents of flavor amino acids, total essential amino acids, total non-essential amino acids, and total amino acids also showed a significant increase in the HES and RA groups compared with the CON group (*p* < 0.05). Furthermore, the HES-RA group exhibited significantly higher contents of flavor amino acids, total essential amino acids, total non-essential amino acids, and total amino acids than those in the HES group or the RA group (*p* < 0.05).

Table 5 presents the free amino acid profile of the serum. Relative to the CON group, significantly higher serum concentrations of arginine, methionine, leucine, cysteine, glutamic acid, and proline were detected in the HES-RA group (*p* < 0.05). The free amino acid profiles were not significantly different between the CON group and the HES or RA groups (*p* > 0.05).

### 3.5. Analysis of Ileal Microbiota

**Figure 2:**
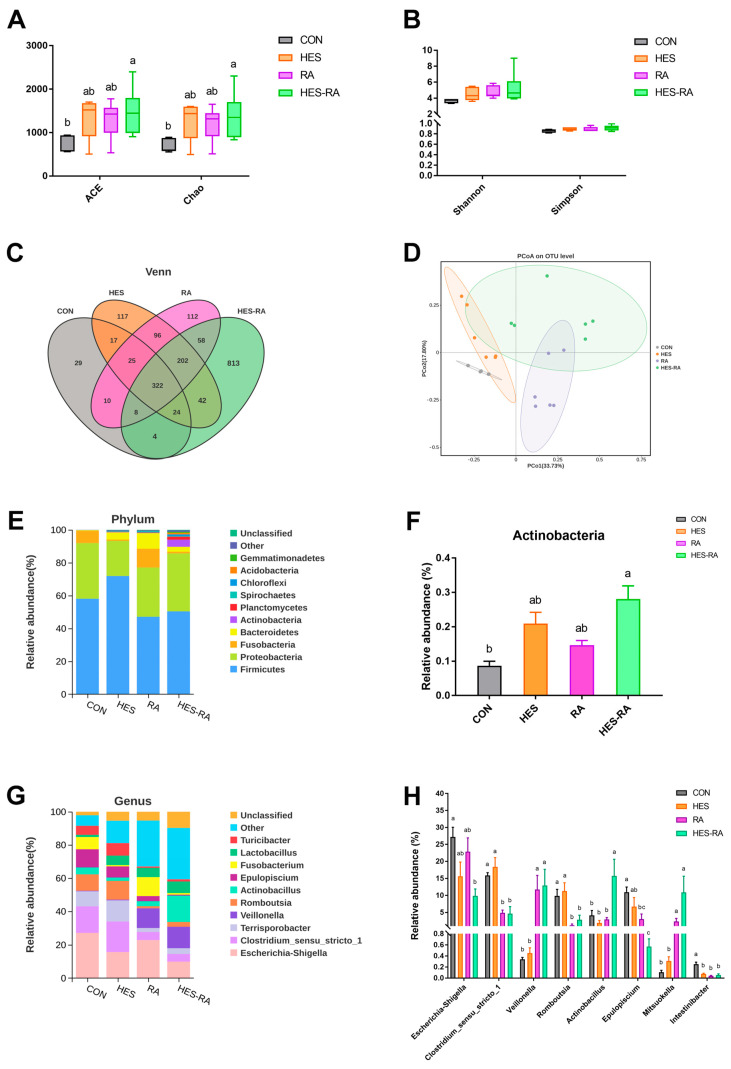
The microbial composition in the ileal digesta of finishing pigs. (**A**) ACE and Chao richness indices. (**B**) Shannon and Simpson diversity indices. (**C**) Venn diagram of core OTUs. (**D**) PCoA ordination based on Bray–Curtis distance with ANOSIM results. (**E**) Relative abundance of the microbial phylum. (**F**) The change in Actinobacteria. (**G**) Relative abundance of the microbial genus. (**H**) The different genera of the ileal digesta microbial. Values represent mean ± SEM (*n* = 6 per group). A significant difference (*p* < 0.05) among different groups is indicated by different lowercase letters. CON = control; HES = hesperidin; RA = rosmarinic acid; HES-RA = hesperidin + rosmarinic acid.

Figure 2 summarizes the shifts in ileal microbial composition of finishing pigs following dietary supplementation with hesperidin and rosmarinic acid. The ACE and Chao richness estimates were significantly elevated in the HES-RA group compared with the CON group (*p* < 0.05; Figure 2A). In contrast, neither Shannon nor Simpson diversity indices differed between the HES-RA and CON groups (*p* > 0.05; Figure 2B). At 97% sequence identity, the OTU clustering identified 439, 845, 833, and 1473 core OTUs in the CON, HES, RA, and HES-RA groups, respectively (Figure 2C). PCoA based on Bray–Curtis distance demonstrated a clear separation among the four groups (*p* < 0.05; Figure 2D).

Firmicutes and Proteobacteria were the predominant phyla across all groups (Figure 2E). A marked enrichment of Actinobacteria was observed in the HES-RA group compared with the CON group (Figure 2F; *p* < 0.05). The phylum-level profiles of the HES and RA groups were not significantly different from that of the CON group (*p* > 0.05).

Genera with a relative abundance exceeding 0.1% are presented in Figure 2G,H. A marked reduction in *Intestinibacter* was observed in the HES group (*p* < 0.05). Relative to the CON group, enrichment of *Veillonella* and *Mitsuokella* was detected in the RA group, accompanied by lower abundances of *Clostridium_sensu_stricto_1*, *Romboutsia*, *Epulopiscium*, and *Intestinibacter* (*p* < 0.05). Significantly higher relative abundances of *Veillonella*, *Actinobacillus*, and *Mitsuokella*, along with lower abundances of *Escherichia_Shigella*, *Clostridium_sensu_stricto_1*, *Romboutsia*, *Epulopiscium*, and *Intestinibacter*, were observed in the HES-RA group compared with the CON group (*p* < 0.05).

### 3.6. SCFA Concentrations

The SCFA concentrations measured in ileal digesta are summarized in Table 6. Relative to the CON group, markedly higher propionate and butyrate levels were detected in the RA group (*p* < 0.05). Acetate, propionate, and butyrate concentrations were likewise elevated in the HES-RA group compared with the CON group (*p* < 0.05). No significant differences in any SCFA measured were found between the HES and CON groups (*p* > 0.05).

### 3.7. Expression of mRNA Related to Protein Synthesis

**Figure 3:**
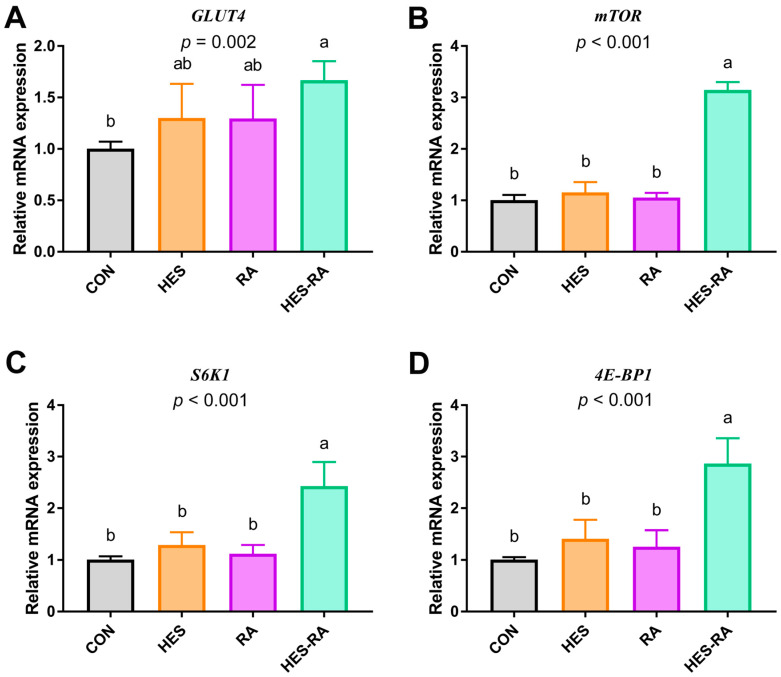
The relative mRNA expressions of protein-related metabolism genes in the Longissimus lumborum muscle of finishing pigs. (**A**) The relative mRNA expression of *GLUT4*. (**B**) The relative mRNA expression of *mTOR*. (**C**) The relative mRNA expression of *S6K1*. (**D**) The relative mRNA expression of *4E-BP1*. Values represent mean ± SEM (*n* = 6 per group). A significant difference (*p* < 0.05) among different groups is indicated by different lowercase letters. CON = control; HES = hesperidin; RA = rosmarinic acid; HES-RA = hesperidin + rosmarinic acid. *GLUT4* = glucose transporter 4; *mTOR* = mammalian target of rapamycin; *S6K1* = ribosomal protein S6 protein kinase 1; *4E-BP1* = eukaryotic initiation factor 4E binding protein 1.

Significantly higher mRNA levels of *GLUT4*, *mTOR*, *S6K1*, and *4E-BP1* were detected in the Longissimus lumborum muscle of the HES-RA group compared with the CON group (Figure 3; *p* < 0.05). No significant changes in the relative mRNA expression of these genes were observed in the HES and RA groups compared to the CON group (*p* > 0.05).

## 4. Discussion

This study provides compelling evidence that this combination of hesperidin and rosmarinic acid (HES-RA) elicits more profound and multifaceted improvements in finishing pig meat quality, compared with the administration of either compound alone. The collective results demonstrate that the HES-RA combination enhances carcass leanness, improves meat quality parameters, and boosts the endogenous antioxidant defense system. In addition, it enriches muscle and serum amino acid profiles, modulates the gut microbiota, and upregulates the mRNA expression of key genes in protein synthesis-related signaling pathways. These findings establish the HES-RA combination as an effective natural dietary strategy for producing high-quality pork. Notably, a companion study from our group has demonstrated that this combined supplementation regimen did not adversely affect growth performance and maintained normal feed efficiency in finishing pigs [11], suggesting that the meat quality improvements were not achieved at the expense of production performance.

The significant improvements in carcass weight, lean meat percentage, and the reduction in backfat depth observed in the HES-RA group indicate a fundamental shift in nutrient partitioning towards protein accretion and away from fat deposition [23]. This combined effect suggests that hesperidin and rosmarinic acid may act on complementary metabolic pathways. Although the individual compounds conferred certain benefits, the combination yielded particularly remarkable effects. The enhanced meat quality parameters, specifically the higher pH45 min, reduced drip loss, and superior meat color (higher a* value, lower L* value), are hallmarks of attenuated post-mortem metabolic stress and oxidative damage [24]. The elevated pH_45min_ is critical, as it indicates a slower decline in muscle pH post-slaughter, resulting from a more gradual glycolysis rate [25]. This is typically associated with improved water-holding capacity, as corroborated by the reduced drip loss [23]. The enhanced color stability (increased redness, reduced lightness) is directly linked to the stability of myoglobin [26]. The potent antioxidant properties of the HES-RA combination likely protect myoglobin from oxidation, thereby preserving the desirable red color. The failure of individual HES or RA treatments to significantly alter these traits underscores the necessity of their combined action, potentially through distinct yet complementary mechanisms that mitigate protein denaturation and lipid oxidation during the critical post-mortem period.

A central finding of this study is the up-regulation of the muscle’s antioxidant system by the HES-RA combination. The significant increases in the activities of GSH-Px, T-SOD, and T-AOC, coupled with a marked reduction in MDA content, provide clear evidence of enhanced oxidative stability. MDA, a terminal product of lipid peroxidation, is a key indicator of oxidative rancidity and quality deterioration [27]. Its reduction is crucial for extending shelf-life and maintaining flavor. The mechanism underlying this antioxidant enhancement may involve the upregulation of the Nrf2 signaling pathway, a master regulator of cellular antioxidant responses [23]. Both hesperidin and rosmarinic acid have been reported to activate Nrf2, promoting its translocation to the nucleus and the subsequent transcription of antioxidant enzymes such as HO-1, GSH-Px, and SOD [28,29]. Although Nrf2 pathway activity was not directly measured in this study, the observed combined effect is consistent with the hypothesis that the combination may induce a more robust or sustained antioxidant response compared to either compound alone. Rosmarinic acid, with its direct free-radical-scavenging capacity attributable to its catechol structure, may serve as a first line of defense, reducing the oxidative load and allowing hesperidin to more effectively up-regulate these endogenous defense mechanisms [28]. This multi-tiered approach combines direct scavenging of free radicals with the up-regulation of endogenous enzyme production. It thereby establishes a highly resilient antioxidant environment within the muscle tissue.

The significant improvements in meat quality observed in the HES-RA group cannot be attributed solely to direct actions on muscle tissue but may also be linked to changes in the gut environment, raising the hypothesis of a potential gut–muscle axis that warrants further investigation. The notable increase in microbial diversity (ACE and Chao indices) and the marked restructuring of the ileal microbial community in the HES-RA group are consistent with the first step of this hypothetical multi-organ communication network. *Escherichia-Shigella*, a common pro-inflammatory enteric pathogen, has been associated with disruption of intestinal barrier integrity and systemic low-grade inflammation [23]. Its reduction in the HES-RA group may reflect a less inflamed gut milieu, which would favor nutrient absorption and reduce the systemic inflammatory tone that negatively impacts muscle protein deposition. *Veillonella* is a well-characterized lactate-utilizing bacterium that produces propionate as a major fermentation end-product, thereby contributing directly to the increased propionate concentrations observed in this group [30]. *Mitsuokella* is likewise a potent propionate and butyrate producer, and its enrichment aligns with the elevated butyrate levels in the HES-RA group [31]. In contrast, the reductions in *Clostridium sensu stricto 1*, *Romboutsia*, and *Intestinibacter*—taxa often prevalent in protein-rich, low-fiber intestinal environments—may reflect a shift in substrate fermentation patterns favoring carbohydrate utilization and SCFA production over putrefactive pathways. Collectively, these taxonomic shifts depict a remodeled ileal ecosystem with enhanced capacity for SCFA biosynthesis, providing a plausible microbial basis for the systemic improvements in meat quality. Of note, the significant increases in acetate, propionate, and butyrate concentrations in the HES-RA group coincided with the improvements in muscle parameters, providing correlative evidence for a possible metabolic link between the gut and muscle tissues [32]. These SCFAs are known to function as signaling molecules that can be absorbed via the portal circulation and exert systemic effects [33]. In particular, butyrate, as a histone deacetylase inhibitor, has been proposed to modulate gene expression in peripheral tissues such as muscle, although this has not been directly tested in the present study [32]. A systemic anti-inflammatory environment potentially induced by SCFAs could theoretically be associated with the observed upregulation of mTOR pathway-related gene expression in muscle tissue. This may inhibit inflammation-mediated protein catabolism and create a metabolic state favorable for protein synthesis [34]. This notion is further supported by the upregulation of GLUT4, suggesting improved glucose uptake efficiency. Moreover, the enhanced antioxidant status in muscle might be partly linked to SCFA-mediated effects, as SCFAs have been reported to enhance systemic antioxidant capacity and alleviate oxidative stress [35]. It is important to emphasize, however, that the supplementation of either compound alone failed to achieve the same degree of microbial modulation or SCFA production, which makes the combined treatment an interesting case, but the causal chain from gut to muscle remains to be experimentally verified. Thus, the observed associations between microbial shifts, SCFA production, and muscle outcomes should be interpreted with caution, as correlation does not necessarily imply causation. Collectively, these data suggest that hesperidin and rosmarinic acid, through their combined influence on the gut microenvironment, could indirectly support muscle health and protein synthesis. Direct evidence for this gut–muscle axis is currently lacking, and these proposed mechanisms require further investigation in targeted studies.

The marked enrichment of free amino acids observed in both the Longissimus lumborum muscle and serum of the HES-RA group represents a notable outcome. The increases in essential amino acids (e.g., lysine, leucine), flavor-enhancing amino acids (e.g., glutamic acid, aspartic acid), and total amino acid content signify an enhancement in the nutritional and sensory value of the pork [36]. This phenomenon can be attributed to two interconnected mechanisms: enhanced protein synthesis and reduced protein degradation [37]. The mTOR pathway integrates signals from nutrients (particularly amino acids) and energy status to initiate translation and regulate protein synthesis [38]. The upregulation of the mRNA expression of key genes in the mTOR anabolic pathway (mTOR, S6K1, 4E-BP1, and GLUT4) suggests an enhanced capacity for muscle protein synthesis. The antioxidant and anti-inflammatory environment created by the HES-RA combination likely reduces oxidative stress-induced inhibition of mTOR signaling. Simultaneously, the improved gut health and potentially enhanced nutrient absorption ensure a better supply of substrates and energy. The increase in muscle arginine, a known activator of mTOR, further supports this anabolic state [39]. This suggests that HES-RA not only protects existing proteins from oxidation but also actively promotes the synthesis of new muscle proteins, leading to an increased deposition of higher-quality protein.

The results of this study support a theoretical model wherein hesperidin and rosmarinic acid act in combination across multiple biological tiers: from modifying the gut ecosystem and its metabolome, to enhancing systemic antioxidant and anti-inflammatory status, and finally to upregulating the expression of anabolic pathway-related genes in muscle tissue. This multi-organ interaction highlights the importance of a holistic nutritional approach to improving meat quality. From a practical standpoint, the use of a combination dose that outperforms higher individual doses offers a cost-effective and efficient strategy for pig production. It aligns with the industry’s need for natural alternatives to antibiotics and synthetic antioxidants to produce pork that meets consumer demands for healthfulness, safety, and quality. Although these interactions remain hypothetical, further research is required to validate the proposed gut–muscle axis under controlled conditions. Future research should focus on elucidating the precise molecular crosstalk between these two polyphenols and validating these findings under large-scale commercial farming conditions. This study has several limitations. The sample size (*n* = 6 per group), although typical for pig nutrition trials, may have limited power to detect subtle effects in microbiota and gene expression analyses. Additionally, only mRNA expression was measured, and the gut–muscle axis remains a hypothesis requiring direct validation. Future studies with larger cohorts and mechanistic designs are warranted.

## 5. Conclusions

Dietary supplementation with hesperidin and rosmarinic acid in combination enhanced antioxidant capacity, optimized gut microbiota and SCFA production, and upregulated the mRNA expression of key genes in the mTOR signaling pathway, and these changes were associated with improved meat quality in finishing pigs.

## Figures and Tables

**Table 1:** Dietary composition and nutrient levels of growing-finishing pigs.

Items	Body Weight (kg)		
	20~50	50~80	80~20
Ingredients (%)			
Corn	68.17	71.70	75.00
Soybean meal	21.96	18.81	16.00
Corn gluten meal	1.00	1.00	1.00
Soybean oil	2.06	1.89	1.50
Limestone	0.63	0.59	0.56
Dicalcium phosphate	1.02	0.98	0.96
NaCl	0.31	0.26	0.26
Premix ^1^	4.00	4.00	4.00
L-Lysine	0.50	0.45	0.43
Methionine	0.10	0.10	0.10
Threonine	0.20	0.18	0.16
Tryptophan	0.05	0.04	0.03
Nutrient levels ^2^			
Digestible energy (MJ/kg)	14.57	14.52	14.41
Crude protein (%)	16.41	15.22	14.23
Ether extract (%)	3.06	3.10	3.14
Calcium (%)	0.63	0.59	0.56
Available phosphorus (%)	0.35	0.33	0.32
Lysine (%)	1.08	0.96	0.88
Methionine (%)	1.21	1.19	1.18
Threonine (%)	0.67	0.61	0.55
Tryptophan (%)	0.18	0.15	0.13

^1^ premix provided per kg of diet: Vitamin A, 1280 IU; Vitamin B1, 1 mg; Vitamin B2, 2 mg; Vitamin B6, 1 mg; Vitamin B12, 0.0048 mg; Vitamin D3, 140 IU; Vitamin E, 10.8 IU; Vitamin K3, 0.48 mg; Niacin, 20 mg; Pantothenic acid, 10 mg; Folic acid, 0.3 mg; Biotin, 0.2 mg; Choline chloride, 200 mg; Fe, 40 mg; Cu, 3 mg; Zn, 40 mg; Mn, 2 mg; I, 0.14 mg; Se, 0.1 mg. ^2^ Values of digestible energy were calculated from data provided by Feed Database in China (2020) (https://www.chinafeeddata.org.cn/admin/Login/slcfb). Crude protein and ether extracts were measured values.

**Table 2:** Predicted means for carcass traits of finishing pigs.

Item	Treatment ^1^				SEM	*p*-Value
	CON	HES	RA	HES-RA		
Carcass weight, kg	86.22 ^b^	88.93 ^b^	90.22 ^b^	95.87 ^a^	7.25	<0.001
Carcass yield, %	74.80	74.94	74.77	73.88	0.24	0.076
Carcass straight length, cm	98.29	98.28	98.37	99.42	0.40	0.162
Carcass oblique length, cm	85.26 ^b^	85.52 ^b^	85.27 ^b^	86.79 ^a^	0.34	0.021
Loin-eye area, cm^2^	47.86	48.16	48.12	49.35	0.39	0.086
Back fat depth, mm	26.73 ^a^	26.43 ^b^	26.28 ^b^	25.68 ^c^	0.04	0.001
Lean meat rate, %	61.04 ^c^	62.13 ^b^	61.79 ^b^	63.58 ^a^	0.19	<0.001
Fat rate, %	18.71 ^a^	18.29 ^a^	18.46 ^a^	16.69 ^b^	0.13	<0.001
Bone rate, %	12.82	12.70	12.98	13.19	0.18	0.262
Skin rate, %	6.98	6.72	6.76	7.16	0.18	0.274

^1^ CON = control; HES = hesperidin; RA = rosmarinic acid; HES-RA = hesperidin + rosmarinic acid. The mean covariate weight used was 90.31 kg. ^a,^^b,c^ Values in the same row with different superscripts significantly differ at *p* < 0.05. Data are expressed as the mean and SEM, *n* = 6.

**Table 3:** The meat quality of finishing pigs.

Item	Treatment ^1^				SEM	*p*-Value
	CON	HES	RA	HES-RA		
pH_45min_	6.18 ^b^	6.18 ^b^	6.20 ^b^	6.32 ^a^	0.01	<0.001
pH_24h_	5.64	5.65	5.64	5.63	0.01	0.603
Meat color L*	42.7 ^a^	42.8 ^a^	42.9 ^a^	41.7 ^b^	0.1	<0.001
Meat color a*	12.6 ^b^	12.6 ^b^	12.5 ^b^	13.5 ^a^	0.1	<0.001
Meat color b*	7.7	7.8	7.8	7.6	0.1	0.761
Drip loss, %	3.65 ^a^	3.74 ^a^	3.93 ^a^	1.88 ^b^	0.18	<0.001
Cooking loss, %	21.78	22.41	22.18	21.11	0.33	0.652
Shear force, N	45.95	46.12	45.96	45.85	0.12	0.938

^1^ CON = control; HES = hesperidin; RA = rosmarinic acid; HES-RA = hesperidin + rosmarinic acid. ^a,b^ Values in the same row with different superscripts significantly differ at *p* < 0.05. Data are expressed as the mean and SEM, *n* = 6.

**Table 4:** The free amino acid profile of the Longissimus lumborum muscle in finishing pigs (mg/g, as-fresh basis).

Item	Treatment ^1^				SEM	*p*-Value
	CON	HES	RA	HES-RA		
EAA						
Lys	15.00 ^b^	17.56 ^ab^	17.51 ^ab^	20.44 ^a^	0.55	<0.001
Met	4.44 ^b^	4.64 ^ab^	4.61 ^ab^	5.05 ^a^	0.08	0.032
Val	8.06	8.30	8.35	9.42	0.20	0.060
Ile	7.62 ^b^	8.52 ^ab^	8.57 ^ab^	8.76 ^a^	0.16	0.045
Leu	15.43 ^b^	17.10 ^ab^	17.18 ^ab^	18.62 ^a^	0.34	0.005
Phe	6.51 ^b^	7.52 ^ab^	7.40 ^ab^	8.24 ^a^	0.18	0.002
His	6.51	6.57	6.61	6.62	0.03	0.518
Thr	8.39 ^b^	9.39 ^ab^	9.26 ^ab^	9.85 ^a^	0.18	0.023
NEAA						
Ala	9.73 ^b^	10.37 ^ab^	10.37 ^ab^	11.77 ^a^	0.27	0.039
Asp	18.18 ^b^	19.40 ^ab^	19.58 ^ab^	20.43 ^a^	0.24	0.004
Glu	27.25 ^b^	27.89 ^ab^	28.02 ^ab^	28.93 ^a^	0.19	0.008
Arg	8.58 ^b^	9.02 ^ab^	9.46 ^ab^	9.98 ^a^	0.19	0.036
Gly	7.05 ^b^	7.35 ^ab^	7.52 ^ab^	8.55 ^a^	0.19	0.021
Ser	6.92 ^b^	7.52 ^ab^	7.79 ^ab^	8.40 ^a^	0.19	0.044
Tyr	6.26 ^b^	6.75 ^ab^	6.84 ^ab^	7.61 ^a^	0.17	0.037
Pro	7.54	7.56	7.55	7.70	0.07	0.820
FAA	70.78 ^c^	74.03 ^b^	74.95 ^b^	79.66 ^a^	0.75	<0.001
TEAA	71.95 ^c^	79.60 ^b^	79.50 ^b^	87.01 ^a^	1.24	<0.001
TNEAA	91.50 ^c^	95.85 ^b^	97.13 ^b^	103.37 ^a^	0.96	<0.001
TAA	163.46 ^c^	175.46 ^b^	176.63 ^b^	190.38 ^a^	2.10	<0.001

Lys = lysine; Met = methionine; Val = valine; Ile = isoleucine; Leu = leucine; Phe = phenylalanine; His = histidine; Thr = threonine; Ala = alanine; Asp = aspartate; Glu = glutamate; Arg = arginine; Gly = glycine; Ser = serine; Tyr = tyrosine; Pro = proline; FAA, flavor amino acids; TEAA, total essential amino acids; TNEAA, total non-essential amino acids; TAA, total amino acids. FAA = Asp + Glu + Gly + Ala + Ser + Pro. ^1^ CON = control; HES = hesperidin; RA = rosmarinic acid; HES-RA = hesperidin + rosmarinic acid. ^a,b,c^ Values in the same row with different superscripts significantly differ at *p* < 0.05. Data are expressed as the mean and SEM, *n* = 6.

**Table 5:** The free amino acid profile of serum in finishing pigs (μmol/L).

Item	Treatment ^1^				SEM	*p*-Value
	CON	HES	RA	HES-RA		
EAA						
Arg	362.76 ^b^	370.30 ^ab^	371.69 ^ab^	396.59 ^a^	4.09	0.011
Lys	112.24	110.64	111.76	110.96	1.26	0.973
Met	45.43 ^b^	46.34 ^ab^	49.61 ^ab^	51.11 ^a^	0.81	0.030
Val	330.84	325.44	326.48	318.59	2.31	0.321
Ile	122.29	124.64	120.26	120.90	1.16	0.582
Leu	206.33 ^b^	209.89 ^ab^	210.11 ^ab^	213.33 ^a^	0.73	0.003
Phe	85.61	86.03	86.63	84.75	0.55	0.697
Thr	130.14	126.76	127.14	128.05	0.85	0.522
NEAA						
Cys	57.55 ^b^	59.59 ^ab^	59.04 ^ab^	64.97 ^a^	1.01	0.038
Ala	231.28	227.04	233.26	221.19	2.35	0.286
Glu	159.01 ^b^	165.22 ^ab^	166.56 ^ab^	172.18 ^a^	1.69	0.040
Gly	980.89	1007.83	984.64	979.27	9.20	0.695
Ser	94.98	94.44	94.18	98.42	0.96	0.387
Tyr	95.71	95.14	95.72	94.89	0.57	0.948
Pro	331.62 ^b^	340.84 ^ab^	337.01 ^ab^	366.12 ^a^	4.44	0.018
TEAA	1395.65	1400.05	1403.68	1424.28	4.48	0.103
TNEAA	1951.04	1990.09	1970.41	1997.04	10.84	0.457
TAA	3346.69	3390.14	3374.09	3421.33	12.00	0.165

Cys = cysteine; for other abbreviations, refer to Table 4. ^1^ CON = control; HES = hesperidin; RA = rosmarinic acid; HES-RA = hesperidin + rosmarinic acid. ^a,b^ Values in the same row with different superscripts significantly differ at *p* < 0.05. Data are expressed as the mean and SEM, *n* = 6.

**Table 6:** The concentrations of SCFAs in the ileal digesta of finishing pigs.

Item	Treatment ^1^				SEM	*p*-Value
	CON	HES	RA	HES-RA		
Acetate, μmol/g digesta	26.12 ^b^	27.13 ^ab^	26.79 ^ab^	27.99 ^a^	0.24	0.035
Propionate, μmol/g digesta	7.02 ^b^	7.25 ^b^	8.06 ^a^	8.43 ^a^	0.14	<0.001
Butyrate, μmol/g digesta	1.89 ^b^	1.95 ^b^	2.33 ^a^	2.37 ^a^	0.06	<0.001
Valerate, μmol/g digesta	0.56	0.57	0.57	0.61	0.01	0.629
Isobutyrate, μmol/g digesta	0.79	0.82	0.82	0.83	0.01	0.771
Isovalerate, μmol/g digesta	0.79	0.83	0.83	0.84	0.01	0.440

^1^ CON = control; HES = hesperidin; RA = rosmarinic acid; HES-RA = hesperidin + rosmarinic acid. a, b Values in the same row with different superscripts significantly differ at *p* < 0.05. Data are expressed as the mean and SEM, *n* = 6.

## Data Availability

Upon request, the corresponding author can provide the data utilized in this work.

## Supplementary Materials

The following supporting information can be downloaded at https://www.mdpi.com/article/10.3390/microorganisms14071518/s1, Table S1: Growth performance of finishing pigs fed diets supplemented with hesperidin and rosmarinic acid. Table S2: Primer sequences used for Real-time quantitative PCR.
