# Supplementary material for: Dietary Supplementation with Hesperidin and Rosmarinic Acid Improves Meat Quality, Modulates Gut Microbiota, and Enhances Antioxidant Capacity in Finishing Pigs
Source: Microorganisms. 2026 Jul 12;14(7):1518. doi: 10.3390/microorganisms14071518 (PMC13413848; doi:10.3390/microorganisms14071518)
Supplement: Supplementary file 1 [file microorganisms-14-01518-s001.zip › microorganisms-4375494-supplementary.pdf]

Table S1. Growth performance of finishing pigs fed diets supplemented with hesperidin and rosmarinic acid.

Item	CON	HES	RA	HES-RA	SEM	*P*-value
ADG (g/d)	850.19^{c}	881.48^{b}	891.48^{b}	975.19^{a}	12.01	<0.001
ADFI (kg/d)	2.79^{c}	2.82^{b}	2.82^{b}	2.95^{a}	0.01	<0.001
F/G	3.28^{a}	3.27^{a}	3.16^{ab}	3.02^{b}	0.03	0.032

CON, control group; HES, hesperidin group; RA, rosmarinic acid group; HES-RA, hesperidin + rosmarinic acid group.

ADG, average daily gain; ADFI, average daily feed intake; F/G, feed-to-gain ratio.

a–c Means within a row with different superscripts differ significantly ($P < 0.05$).

Data are reproduced from our previous study [1].

Reference

[1] ZHAO, W., WU, J., DANG, M., WANG, J., & ZHU, W. (2023). Combination of hesperidin and rosmarinic acid affects cecal morphology, antioxidant function, microbiota structure, and barrier function of finishing pigs. Acta Microbiologica Sinica, 63(11), 4356-4371. https://doi.org/10.13343/j.cnki.wsxb.20230225 (in Chinese)

Table S2. Primer sequences used for Real-time quantitative PCR

Genes	Forward primer (5'-3')	Reverse primer (5'-3')	Accession number
GLTU4	CAACCAGCCTATGCCACCATCG	GCCCGTTCCACCAGCAACAC	NM_001128433.1
mTOR	AGTACCTCCAGGACACCATGAACC	CAGACCTCACAGCCACAGAAAGC	XM_003127584.6
S6K1	TCAGTGGTGGGAACGACAAACAAG	ACTCAGTAGCAGGCGGACTCG	XM_005660083.2
4E-BP1	TCGGAACTCACCTGTGACCAAAAC	GTGGTTCTGGCTGGCATCTGTG	NM_001244225.1
GAPDH	CAAGGCTGTGGGCAAGGTCATC	TTCTCCAGGCGGCAGGTCAG	NM_001206359.1

GLUT4 = glucose transporter 4; *mTOR* = mammalian target of rapamycin; *S6K1* = ribosomal protein S6 protein kinase 1; *4E-BP1* = eukaryotic initiation factor 4E binding protein 1; *GAPDH* = glyceraldehyde phosphate dehydrogenase.
